# Identification of Abietane-Type Diterpenoids and Phenolic Acids Biosynthesis Genes in *Salvia apiana* Jepson Through Full-Length Transcriptomic and Metabolomic Profiling

**DOI:** 10.3389/fpls.2022.919025

**Published:** 2022-06-08

**Authors:** Jiadong Hu, Feiyan Wang, Fengying Liang, Ziding Wu, Rui Jiang, Jinxing Li, Junfeng Chen, Shi Qiu, Jing Wang, Yuchen Zhang, Qing Li, Wansheng Chen

**Affiliations:** ^1^Center of Chinese Traditional Medicine Resources and Biotechnology, Institute of Chinese Materia Medica, Shanghai University of Traditional Chinese Medicine, Shanghai, China; ^2^Department of Pharmacy, Second Affiliated Hospital of Naval Medical University, Shanghai, China

**Keywords:** *Salvia apiana* Jepson, biosynthesis, transcriptomics, metabolomics, phenolic acids, abietane-type diterpenoids

## Abstract

*Salvia apiana* (*S. apiana*) Jepson is a medicinal plant that is frequently used by the Chumash Indians in southern California as a diaphoretic, calmative, diuretic, or antimicrobial agent. Abietane-type diterpenoids (ATDs) and phenolic acids (PAs) are the main bioactive ingredients in *S. apiana*. However, few studies have looked into the biosynthesis of ATDs and PAs in *S. apiana*. In this study, using metabolic profiling focused on the ATDs and PAs in the roots and leaves of *S. apiana*, we found a distinctive metabolic feature with all-around accumulation of ATDs, but absence of salvianolic acid B. To identify the candidate genes involved in these biosynthesis pathways, full-length transcriptome was performed by PacBio single-molecule real-time (SMRT) sequencing. A total of 50 and 40 unigenes were predicted to be involved in ATDs and PAs biosynthesis, respectively. Further transcriptional profile using Illumina HiSeq sequencing showed that the transcriptional variations of these pathways were consistent with the accumulation patterns of corresponding metabolites. A plant kingdom-wide phylogenetic analysis of cytochromes (CYPs) identified two CYP76AK and two CYP76AH subfamily genes that might contribute for the specific ATDs biosynthesis in *S. apiana*. We also noticed that the clade VII laccase gene family was significantly expanded in *Salvia miltiorrhiza* compared with that of *S. apiana*, indicating their involvements in the formation of salvianolic acid B. In conclusion, our results will enable the further understanding of ATDs and PAs biosynthesis in *S. apiana* and *Salvia* genus.

## Introduction

*Salvia apiana* (*S. apiana*) Jepson, commonly known as white sage or bee sage, belongs to the genus *Salvia* L. (Lamiaceae: Nepetoideae: Mentheae: Salviinae). *S. apiana* is specific for California's and Baja California's flora (Will and Claßen-Bockhoff, 2017). It is widely distributed in the California Floristic Province and forms the chaparral and desert sage community together with other eighteen members of Salvia sections such as Echinosphace and Audibertia (Walker et al., 2015). Therefore, its taxonomical position is quite essential for understanding the phylogenetic relationship and origin of genus *Salvia* in the New World. However, the available nuclear genes in *S. apiana* are very limited, which hamper its phylogenetic studies based on molecular data.

The application history of *S. apiana* is unique due to its distribution area. It is widely used by the Chumash Indians as diaphoretic, calmative, diuretic, and antimicrobial agent, as well as a burning sage used in religious practices (called khapshikh or xapcix) (Walker et al., 2015; Krol et al., 2021). In modern times, *S. apiana* is demonstrated to have pharmacological activities, including antimicrobial, anti-inflammatory, gamma-aminobutyric acid (GABA)ergic, analgesic, antioxidant, cytotoxic, and antitumor activities, owing to its special chemical constitution (Khan et al., 2016; Saeed et al., 2016; Srivedavyasasri et al., 2017; Afonso et al., 2019; Krol et al., 2021). Previous phytochemical studies on *S. apiana* have identified substantial amounts of essential oil, accompanied by a variety of triterpenes, C23 terpenoids, diterpenes, flavonoids, and phenolic acids (PAs) (Krol et al., 2021). A distinctive metabolic feature of *S. apiana* is that it can produce abietane-type diterpenoids (ATDs) in the aerial part of the plant, while the other American *Salvia* species can only produce clerodane derivatives (Dentali, 1991; Bisio et al., 2019; Krol et al., 2021). Carnosic acid and its derivatives, such as 16-hydroxycarnosic acid, rosmanol, and 16-hydroxycarnosol, etc., constituted the majority of this chemical group (Luis et al., 1996). Tanshinones, such as tanshinones IIA, tanshinones B, and cryptotanshinone, represent another group of ATDs, are usually regarded as the feature compounds in the East Asia taxa of *Salvia* genus (Guo et al., 2016; Ma et al., 2021). Interestingly, cryptotanshinone was also found in the root of *S. apiana* (González et al., 1992), indicating the presence of other tanshinones chemicals and a much more complex metabolic composition of diterpenes in *S. apiana* as well.

Owing to the restricted distribution range and uncontrolled harvesting, *S. apiana* is now at high risk of rapid decline or extinction (Adlof, 2015). Therefore, studies concerning botany, cultivation, cell culture, and metabolic engineering of *S. apiana* are necessary for efficient propagation of natural resources and production of bioactive metabolites, which largely rely on the understanding of metabolic pathways of these bioactive compounds and the genetic resource at both the transcriptomic and genomic levels (D'Amelia et al., 2017). To date, studies of *S. apiana* have mainly focused on its chemical compositions and biological activities of metabolites. However, little about the genetics of the plant was concerned, which has resulted in the lack of knowledge about the biosynthesis of several pharmacologically active chemical groups in *S. apiana*.

Here, we used single-molecule real-time (SMRT) sequencing on the PacBio Sequel platform to obtain full-length transcriptome information of *S. apiana*. A plant metabolomics and chemometrics analysis based on ultra-high-performance liquid chromatography coupled with quadrupole time-of-flight mass spectrometry (UHPLC-Q-TOF-MS/MS) was further applied to characterize the metabolic profiling and screen the chemical markers corresponding to underground and aerial parts of *S. apiana*. Based on the metabolic profiling of ATDs and PAs, the transcriptional patterns of genes in diterpenoids and phenolic acid biosynthesis pathways were elucidated in *S. apiana* using Illumina sequencing.

## Results

### Metabolic Profiling of *S. apiana*

**Figure 1:**
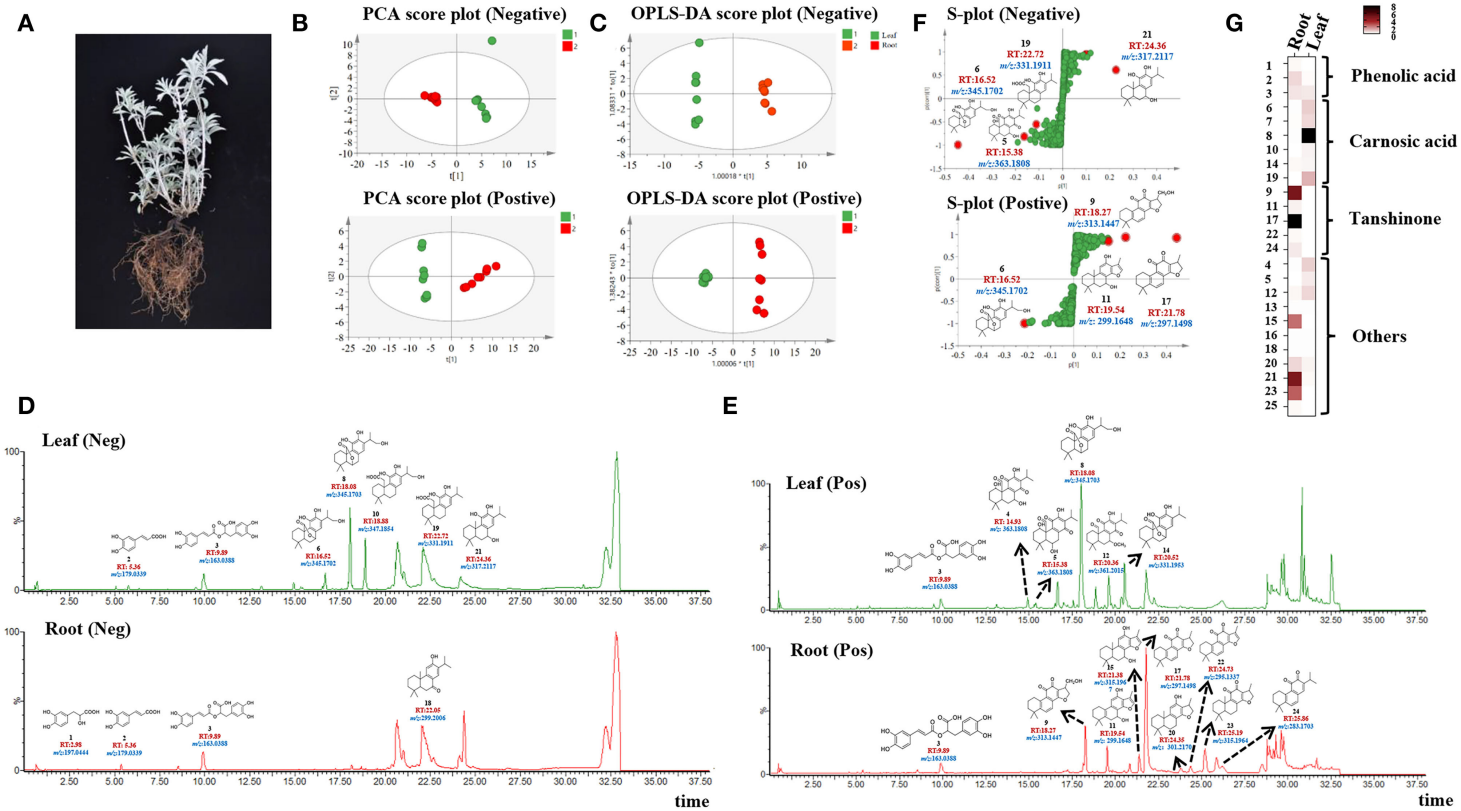
Metabolomics and chemometric analyses of extracts from roots and leaves by QTOF-MS in *Salvia apiana*. **(A)** Image of whole plant of *S. apiana*. **(B)** PCA analysis of roots and leaves in the negative-ion and positive-ion modes. **(C)** OPLS-DA analysis of roots and leaves in the negative-ion and positive-ion modes. **(D)** Total ion current (TIC) chromatograms of leaves and roots in the negative-ion modes annotated with identified metabolites (1, 2, 3, 6, 8, 10, 18, 19 and 21), including corresponding structure, retention time (RT), and mass to charge ratio (m/z). **(E)** Total ion current (TIC) chromatograms of leaves and roots in the positive-ion modes annotated with identified metabolites (3, 4, 5, 8, 9, 11, 12, 14, 15, 17, 20, 23 and 24), including corresponding structure, retention time (RT), and mass to charge ratio (m/z). **(F)** S-plot derived from OPLS-DA analysis in the negative-ion and positive-ion modes annotated with the most distinct metabolites, including corresponding structure, retention time (RT), and mass to charge ratio (m/z). **(G)** Relative content of identified metabolites in roots and leaves. 1, danshensu; 2, caffeic acid; 3, rosmarinic acid; 6, 16-oxhydryl-carnosol; 7, rosmanol; 8, 16-oxhydryl-isocarnosol; 9, 17-oxhydryl-cryptotanshinone; 10, 6-oxhydryl-carnosic acid; 13, 11, 20-dihydroxysugiol; 14, carnosol; 16, 11-hydroxysugiol; 17, cryptotanshinone; 18, sugiol; 19, carnosic acid; 22, tanshinone IIA; 24, miltirone; 25, 11-hydroxyferruginol. Names of metabolite 4, 5, 11, 12, 15, 20, 21 and 23 were listed in Supplementary Table 1.

To have a thoroughly chemical constitutes of ATDs and PAs, ultra-high-performance liquid chromatography coupled with quadrupole time-of-flight mass spectrometry (UPLC-Q-TOF-MS) was applied to samples from the aerial (leaves) and underground (roots) parts of *S. apiana* separately (Figure 1A). Peak detection, alignment, and normalization were performed by MS-DIAL in both the negative and positive ion modes. Using principal component analysis (PCA) and orthogonal partial least-squares discriminant analysis (OPLS-DA), we found that the leaf and root groups were significantly separated from each other in both the negative and positive ion modes (Figures 1B,C). Among these metabolites, ATDs and PAs were focused for further characterization. Twenty-five metabolites were identified in total based on accurate mass and MS/MS fragment patterns, eight of which were identified using chemical standards and the rest were discovered by analyzing the tR, MS fragment data, and comparing it to literatures (Figures 1D,E, Supplementary Table 1 and Supplementary Figures 1A,B). As a result, carnosic acid (19) and its derivatives (6, 7, 8, and 10), along with the common precursors of ATDs such as sugiol (10), 11-hydroxysugiol (11), and 11,20-dihydroxysugiol (13), were discovered in leaves (13), while tanshinones (17, 22, 24, and 25), another group of ATDs, were specially detected in roots. In addition, ATD precursors (10, 11, and 13) were also found in roots. Furthermore, PAs as danshensu (1), caffeic acid (2), and rosmarinic acid (3) were found. The S-plots produced by the OPLS-DA analysis identified the most distinct metabolites [with variable importance in projection (VIP) value >4, Supplementary Tables 2, 3] (Figure 1F). As expect, ATDs as compound 5, 6, 11, 17, 19, and 21 were the most distinct metabolites between roots and leaves (Figure 1G). Notably, *S. apiana* was found to contain both the tanshinones- and carnosic acid-related ATDs, which are quite unique in *Salvias*. Since *Salvia miltiorrhiza* (*S. miltiorrhiza*), for example, just contains tanshinones in roots, while *Salvia officinalis* produces only carnosic acid and related compounds in leaves (Guo et al., 2013; Ignea et al., 2016; Scheler et al., 2016).

### Full-Length Sequencing and Annotation of *S. apiana* Transcriptome

**Figure 2:**
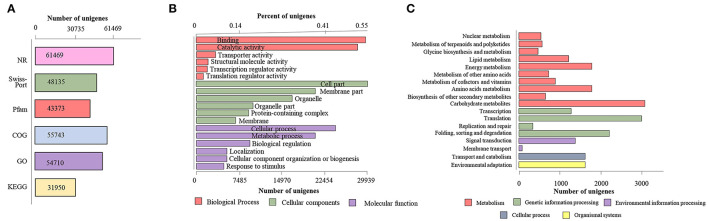
General annotation of *S. apiana* transcriptome. **(A)** Annotation statistics searching against different data banks. **(B)** The most enriched Gene Ontology (GO) terms in three major functional categories. The top six terms of each category were represented. **(C)** The most enriched Kyoto Encyclopedia of Genes and Genomes (KEGG) pathways. Different databases, the GO categories, and the KEGG pathways are distinguished by colors.

The full-length transcriptome of *S. apiana* was obtained using PacBio SMRT sequencing. A total of 36,953,062 subreads (39.30 Gb) were produced, with the average length of 1,040 bp (Table 1). Circular consensus long-read sequencing was further performed to generate highly accurate long high-fidelity (HiFi) reads to improve the quality of the subreads. As a result, a total of 310,713 circular consensus sequences (CCSs) were generated, with an average length of 1,317 bp and the N50 of 1,565 bp (Table 1). The full-length non-chimeric (FLNC) reads were identified by screening the coexistence of 5′-primers, 3′-primers, and poly-A tails, which resulted in 189,999 FLNC reads (61.15% of CCS) with an average length of 894 bp and the N50 of 1,255 bp (Table 1). After clustering the redundant data, 110,985 final consensus reads were obtained, among which 44,169 (39.80%) consensus reads were over 1,000 bp, 8,522 (19.29%) consensus reads were over 2,000 bp, and 652 (7.65%) consensus reads were over 3,000 bp in length (Figure 2).

**Table 1:** Statistics of *S. apiana* single-molecule real-time (SMRT) sequencing results.

**Project**	**SMRT**
Total subreads	36,953,062
Total subread nucleotides (bp)	39,303,737,421
CCS reads	310,713
FLNC reads	189,999
Consensus reads	110,985
Representative transcripts	78,009
Total size (bp) of representative transcripts	81,125,619
GC content	0.44
Transcript length range (bp)	51–6,262
Average transcript size (bp)	1,039.95
Median transcript size (bp)	910
N50 transcript size	1,466
Transcripts with functional annotation	61,659
Nr	61,469
Swiss-Prot	48,135
Pfam	43,373
KEGG (KO)	31,950
COG	55,743
GO	54,710

Gene annotation was carried out by searching against six protein databases [nonredundant (NR), SwissProt, Pfam, Kyoto Encyclopedia of Genes and Genomes (KEGG), Gene Ontology (GO), and Clusters of Orthologous Groups of proteins (COG)] based on sequence similarities. A total of 61,659 (79.04% of 78,009) transcripts were mapped to at least one database, with NR having the highest number of hits (61,469 transcripts, 78.80%, Table 1 and Figure 2A). Among these transcripts, 54,710 (70.13%) transcripts were linked to the GO categories, including molecular functions (MFs), cellular components (CCs), and biological processes (BPs) (Figure 2B), with CCs as the majority of the GO terms. By the KEGG, 31,950 transcripts were further assigned into 135 biological pathways (Figure 2C and Supplementary Table 4). Notably, a total of 581 transcripts were sitting in the “metabolism of terpenoids and polyketides” pathway, which would help to reveal the ATDs biosynthesis pathways in *S. apiana* in the future.

### Phylogenetic Analysis of *S. apiana*

**Figure 3:**
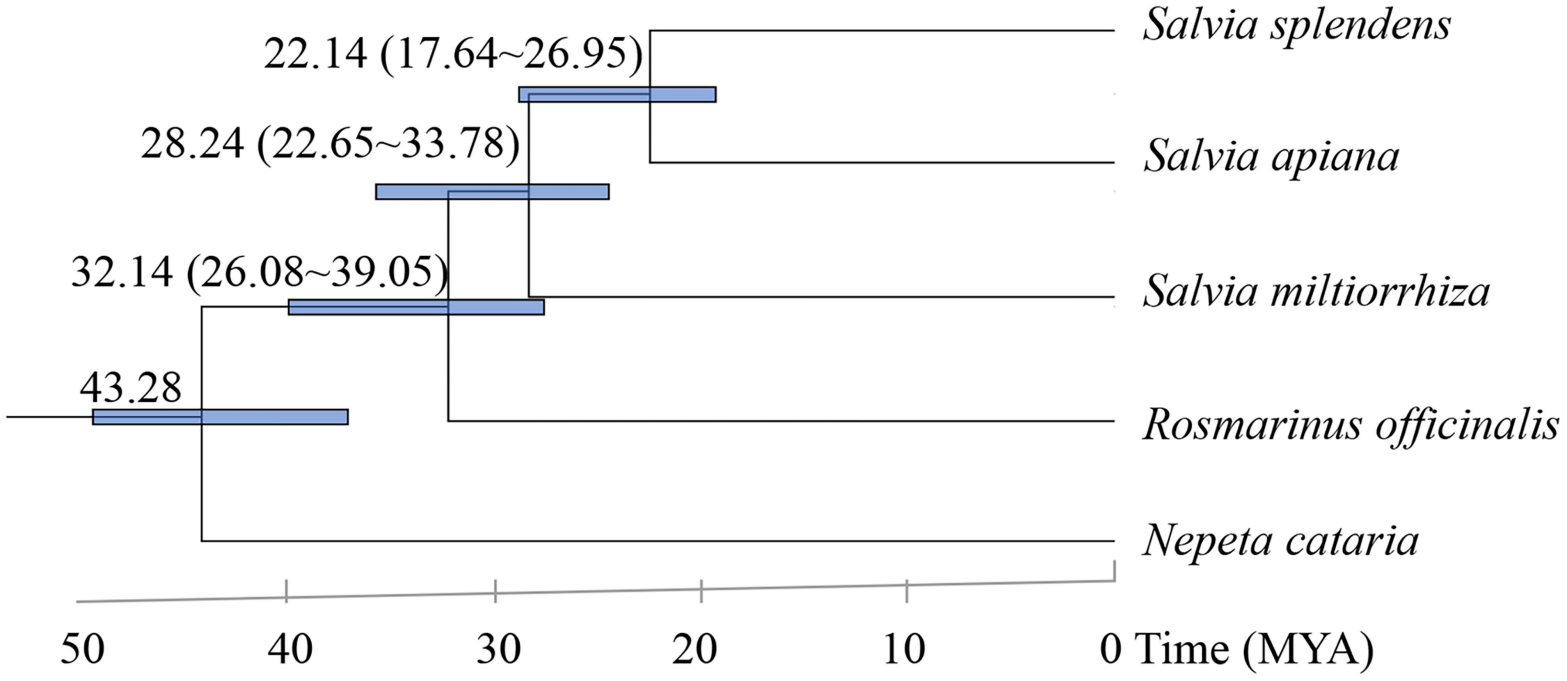
Phylogenetic tree of five plant species. Blue numerical value beside each node shows the estimated divergence time of each node (MYA, million years ago).

To determine the phylogenetic position of *S. apiana* within the genus *Salvia*, orthologous genes from other available *Salvia* genomes [*Salvia splendens* (*S. splendens*), *S. miltiorrhiza*, and *Rosmarinus officinalis*], with *Nepeta cataria* (Nepeta, Lamiaceae) as an outgroup (Dong et al., 2018; Bornowski et al., 2020; Lichman et al., 2020b; Song et al., 2020), were annotated. In total, 130 single-copy genes were selected and used to reconstruct a maximum likelihood phylogenetic tree (Figure 3). As expected, *S. apiana* was phylogenetically categorized into the same taxa as *S. splendens* in America, which might diverge from *S. miltiorrhiza*, a species of East Asia taxa, at about 28.24 Mya.

### Transcriptome Profiling of *S. apiana* Leaves and Roots

**Figure 4:**
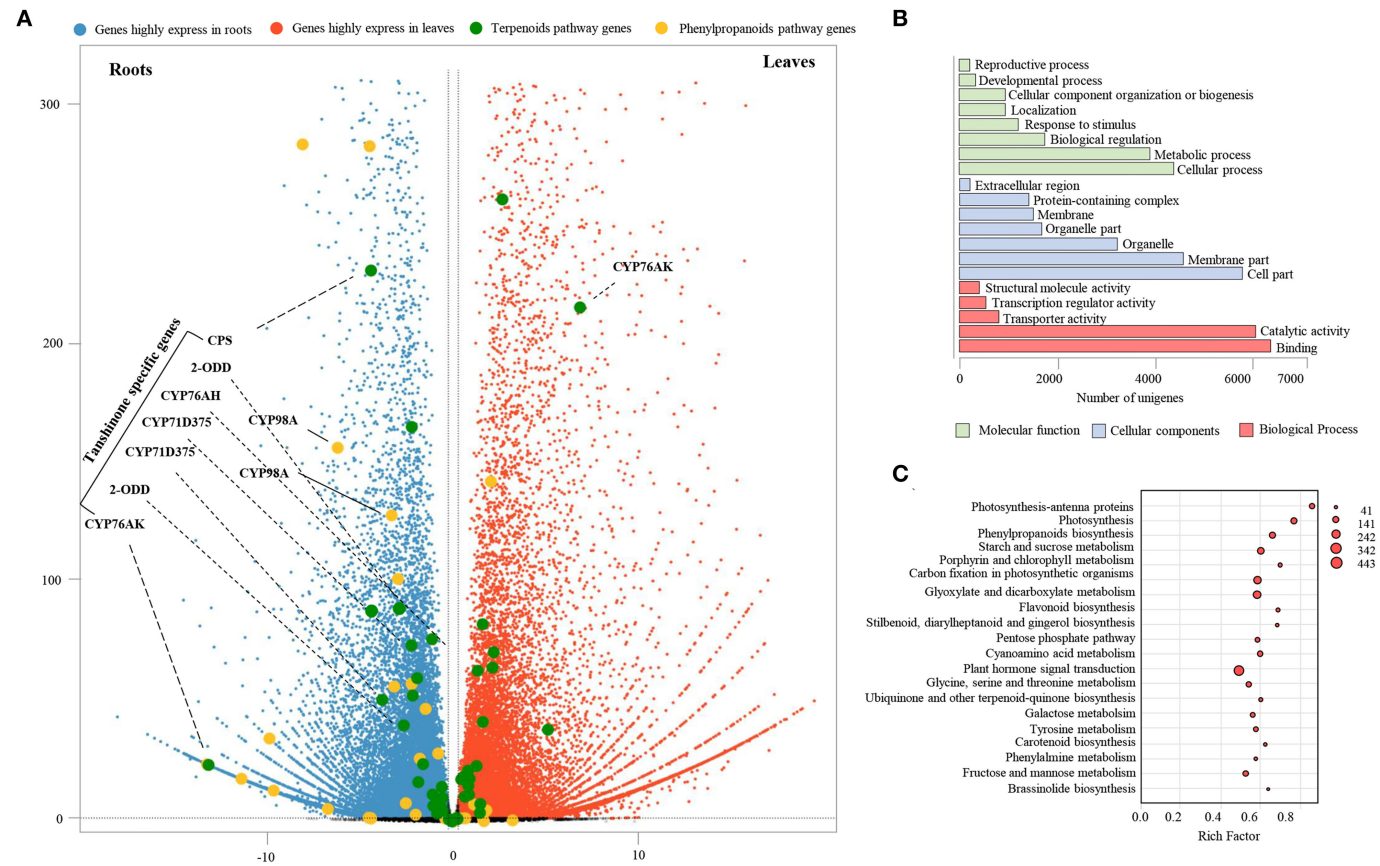
Transcription profiles of *S. apiana* roots and leaves. **(A)** Volcano plots represent the differentially expressed genes (DEGs) with higher transcriptional level in roots (blue plots) and leaves (red plots), respectively. The genes involved in terpenoids (green plots) and phenolic acids (yellow plots) biosynthesis are marked. **(B)** Statistical analysis of the most enriched GO terms from three major categories. **(C)** The top twenty KEGG pathways with the most significant variations of DEGs between the roots and leaves. The number of DEGs is represented by the size of circle.

To identify differentially expressed genes (DEGs) in leaves and roots of *S. apiana*, RNA sequencing (RNA-seq) was performed on the Illumina NovaSeq 6000 platform. Three samples of each organ were sequenced, which produced 42.97 Gb clean data in total (Supplementary Table 5). After assembly, unigenes derived from Illumina sequencing were mapped to full-length transcriptome datasets with a match percentage ranging from 71.81 to 79.01%. Using DEGs analyses, we showed that the transcriptional levels of 15,152 and 14,954 transcripts were significantly higher in roots and leaves, respectively (Figure 4A). To figure out the most significant functional difference between roots and leaves, the GO enrichment of DEGs was further performed (Figure 4B). As a result, metabolic process and cellular process were the most enriched terms in the category of molecular function. In the category of cellular components, most DEGs were enriched in membrane part and cell part. In the category of cellular components, catalytic activity and binding were the most different terms. We noticed that in terms of metabolic process and catalytic activity, these were massively enriched, suggesting that the secondary metabolic pathways in roots and leaves were significantly different. Consistent with the physiological function differences between the aerial and underground parts of plants, photosynthesis, primary metabolic, and plant hormones were found as the most different pathways by the KEGG pathway analysis. In the secondary metabolic pathways, phenylpropanoids biosynthesis was the most significant pathway, showing different transcriptional levels of lignins, flavonoids, and phenolic acids. The variation in diterpenoids pathways was not substantial, which could be attributed to the fact that different metabolites share similar upstream pathways (Figure 4C).

### Transcriptional Variation in Abietane-Type Diterpenoids and Phenolic Acids Biosynthetic Pathways

**Figure 5:**
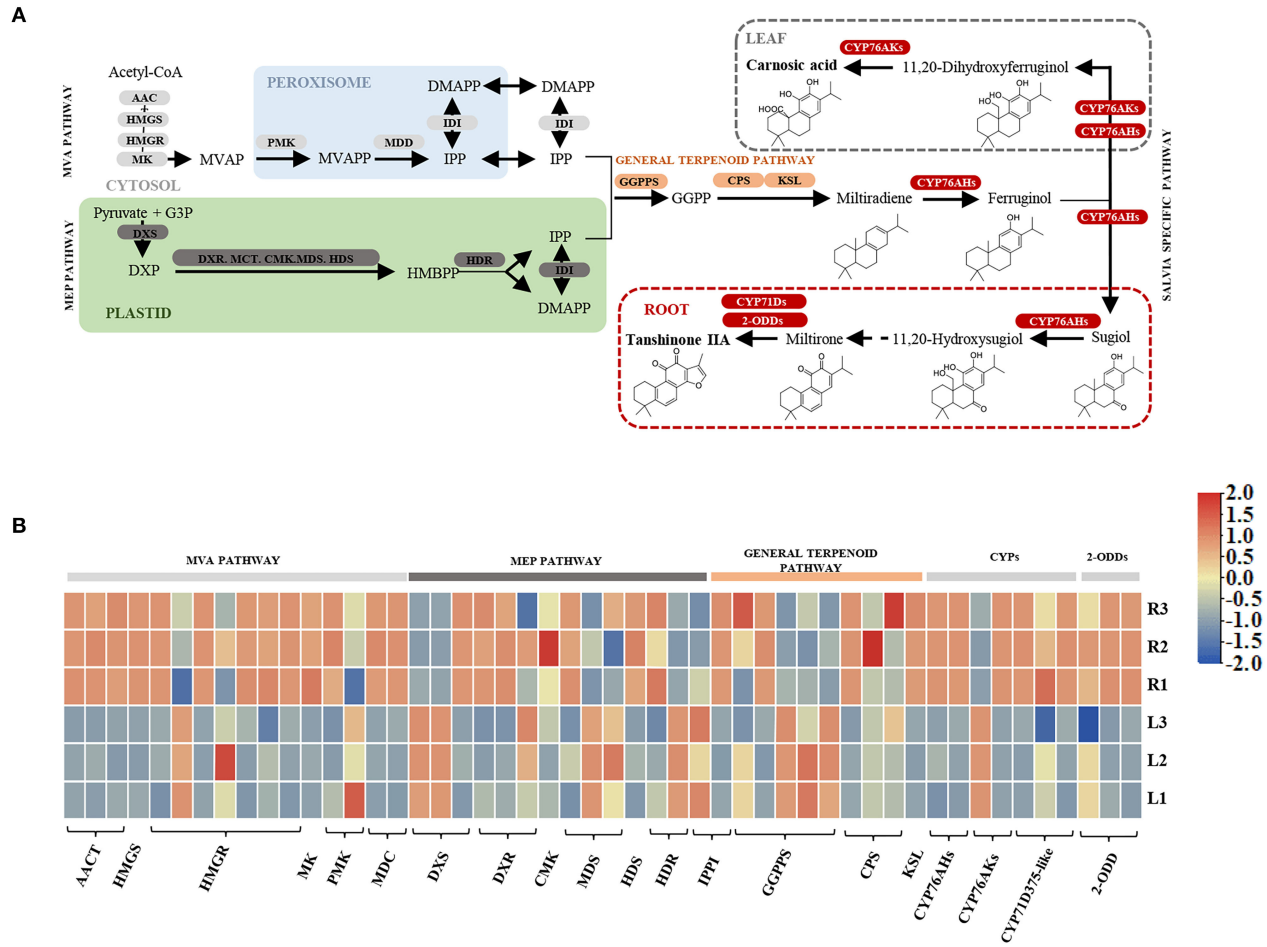
A proposed biosynthetic pathway of abietane-type diterpenoids (ATDs) in *S. apiana*. **(A)** Pathways and genes involved in the ATDs biosynthesis. **(B)** Transcription profile of candidate ATDs biosynthetic genes in roots and leaves of *S. apiana*. Three biological replicates from the leaf (L) and root (R) were plotted individually. AACT, Acetyl-CoA acetyltransferase activity; CYP, cytochrome P450; 2-ODD, 2-oxoglutarate-dependent dioxygenase; HMGS, 3-hydroxy-3-methylglutaryl coenzyme A synthase; HMGR, hydroxymethylglutaryl-CoA reductase; KSL, kaurene synthase-like enzymes; MK, mevalonate phosphotransferase; MVAP, mevalonate 5-phosphate; PMK, phosphomevalonate kinase; MVAPP, mevalonate 5-diphosphate; MDD, mevalonate diphosphate decarboxylase; IPP, isopentenyl diphosphate; IDI, isopentenyl diphosphate delta-isomerase; DMAPP, dimethylallyl diphosphate; DXS, 1-deoxy-D-xylulose-5-phosphate synthase; DXR, 1-deoxy-D-xylulose-5-phosphate reductoisomerase; MCT, 2-C-methyl-D-erythritol 4-phosphate cytidylyltransferase; CMK, 4-(cytidine 50-diphospho)-2-C-methyl-D-erythritol kinase; MDS, 2-C-methyl-D-erythritol 2,4-cyclodiphosphate synthase; HDS, 4-hydroxy-3-methylbut-2-en-1-yl diphosphate synthase; HDR, 1-hydroxy-2-methyl-2-(E)-butenyl 4-diphosphate reductase; GPPS, geranyl pyrophosphate synthase; GPP, geranyl diphosphate; GGPP, geranylgeranyl diphosphate; MVA, schematic of mevalonate; MEP, 2-C-methyl-D-erythritol 4-phosphate/1-deoxy-D-xylulose 5-phosphate.

**Figure 6:**
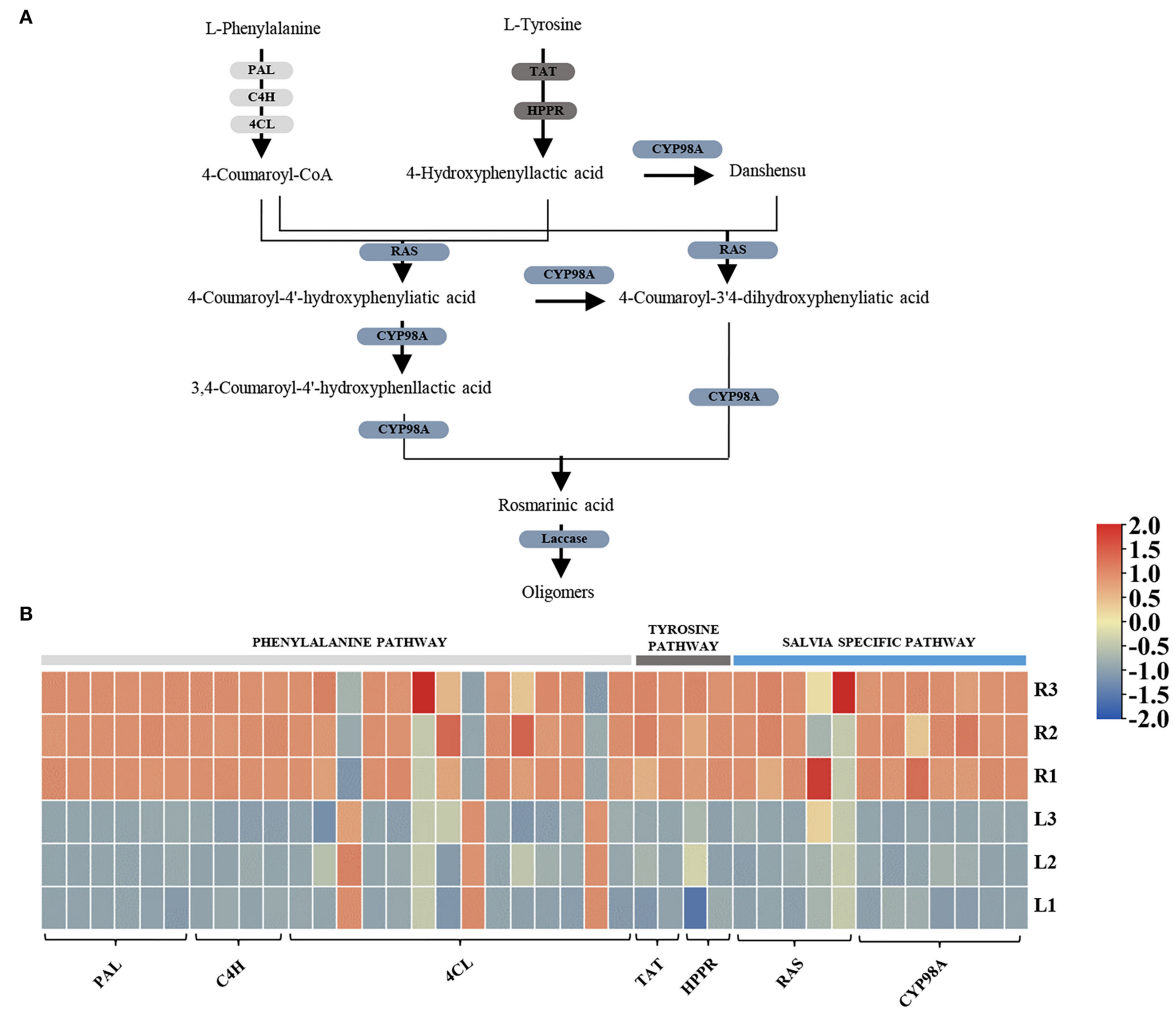
A proposed biosynthetic pathway of phenolic acids (PAs) in *S. apiana*. **(A)** Pathways and genes involved in PAs biosynthesis. **(B)** Expression profile of candidate PAs biosynthetic genes. Three biological replicates from the leaf (L) and root (R) were plotted individually. PAL, phenylalanine ammonia lyase; C4H, cinnamate 4-hydroxylase; 4CL, 4-coumarate:coenzyme A ligase; TAT, tyrosine aminotransferase; HPPR, hydroxyphenylpyruvate reductase; RAS, rosmarinic acid synthase; CYP, cytochrome P450.

As the KEGG pathway analysis showed above, metabolism of terpenoids and polyketides (KEGG Ortholog 09109) represented as one of the most enriched pathways with 581 unigenes (Supplementary Table 6). According to the elucidated ATDs pathway in other *Salvia* plants, including *S. miltiorrhiza, S. fruticose*, and *S. officinalis* (Guo et al., 2013; Ignea et al., 2016; Scheler et al., 2016), we predicted the ATD biosynthesis pathway in *S. apiana* (Figure 5A). A total of 50 unigenes were supposed to be involved in the ATDs biosynthesis and could be considered as four modules. The geranylgeranyl diphosphate (GGPP) synthesis via the methylerythritol 4-phosphate (MEP) and mevalonate (MVA) pathways, along with the general diterpenoids catalytic steps, provided the common precursor (ferruginol) for ATDs biosynthesis. Then, the pathway was split into the biosynthesis of carnosic acids and tanshinones catalyzed by different CYP76AHs and CYP76AKs, respectively. The transcriptional level of these genes was further profiled by DEG analysis (Figure 5B). Most genes in MVA pathway showed high transcriptional levels in root, indicating that they play a prominent role in the production of the terpenoids precursor, isopentenyl diphosphate (IPP). However, the genes in MEP pathway and ferruginol synthesis did not show significant transcription trend. In addition, most *CYP76AH, CYP76AK*, and *2-ODD* genes, which were specific for ATDs biosynthesis, showed higher transcriptional level in root, indicating the high accumulation of tanshinones. There was also one *CYP76AK* gene (transcript_30977) expressed highly in leaf, which might contribute to the carnosic acids biosynthesis. Taken together, the transcriptional profile of ATDs pathway was basically consistent with its metabolic properties in roots and leaves of *S. apiana*. Additionally, forty unignes might be involved in the biosynthesis of PAs were annotated (Figure 6A), most of which were highly expressed in root, consisting with the high accumulation of phenolic acids in roots (Figure 6B). Given that different ATDs and PAs are distributed in roots and leaves, common genes involved in both the metabolic pathways, especially their upstream genes, were found to be highly expressed in both the organs (Figure 4A). Genes related to the metabolic branches, on the other hand, were often organ specific. For example, genes in tanshinones biosynthesis (*CPS, CYP76AHs, CYP76AKs, CYP71D375s*, and *2-ODDs*) were mostly highly expressed in roots.

### Phylogeny of Cytochrome and Laccase Families

**Figure 7:**
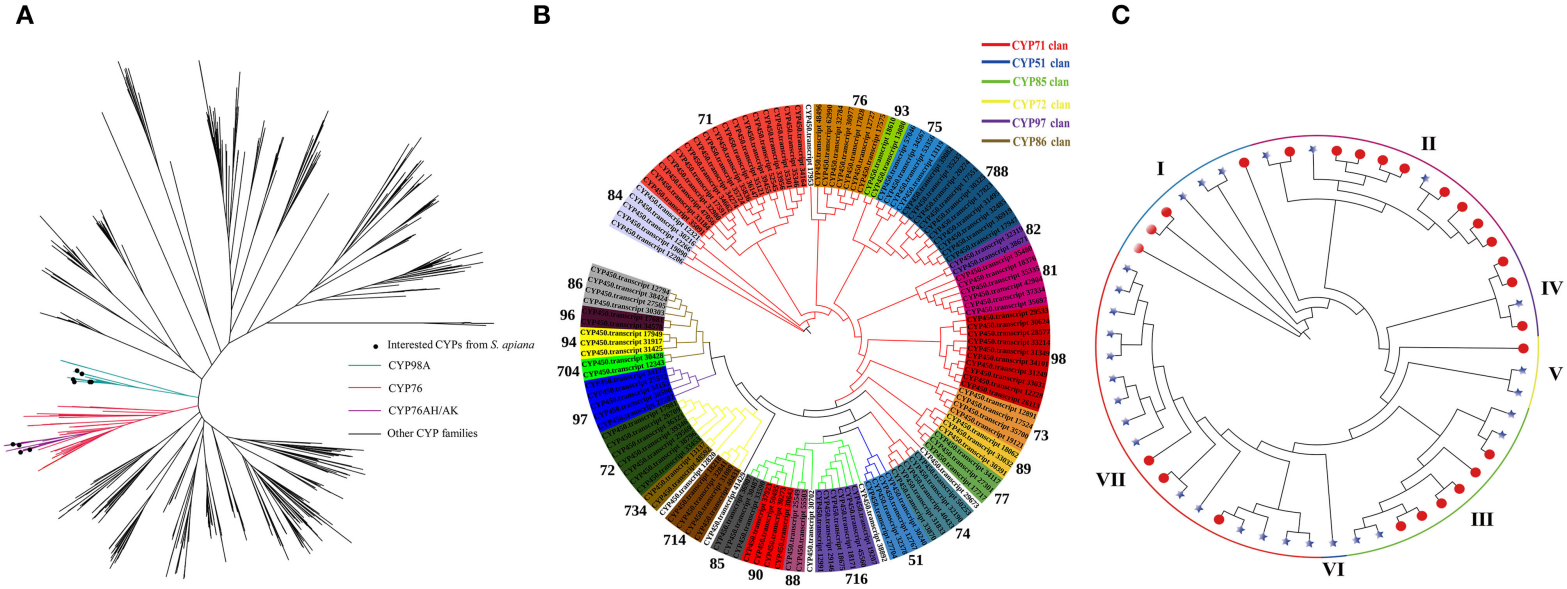
Phylogenetic analysis of cytochrome (CYP) and laccase gene families from *S. apiana*. **(A)** Maximum likelihood (ML) phylogenetic tree of 132 CYPs from *S. apiana* compared to 578 CYPs from other plant species. The blue branch represents the CYP98A subfamily. The red branch represents the CYP76 family, with CYP76AK and CYP76AH subfamilies in purple. **(B)** Maximum likelihood (ML) phylogenetic tree of 132 CYPs from *S. apiana*. Branches in different colors represent different CYP clans: CYP71 (red), CYP51 (blue), CYP85 (green), CYP72 (yellow), CYP97 (purple), and CYP86 (brown). Subfamilies were indicated by background colors. **(C)** ML phylogenetic tree of laccases from *S. apiana* (asterisks) and *Salvia miltiorrhiza* (*S. miltiorrhiza*) (red nodes).

The functional specificity of Lamiaceae cytochromes (CYPs) determines the particular products of ATDs and PAs in *Salvia* plants (Hansen et al., 2021). Therefore, a total of 132 *CYP* genes were discovered in *S. apiana* by Pfam annotation and used for phylogenetic tree reconstruction, together with 578 CYPs from other plants (Figure 7A). Among these, CYP98A and CYP76 families were grabbed for detailed analysis. CYP98As are usually related to phenolic acids biosynthesis, while CYP76AKs and CYP76AHs are unique in Lamiaceae, which regulate the specific ATDs biosynthesis. In *S. apiana*, we found six homologous of CYP98A, two homologous each of CYP76AK and CYP76AH, respectively. We further studied the phylogeny of all the CYPs from *S. apiana*, which were classified into 26 families among six clans (Figure 7B), with 71 clan representing the richest families and CYP71 as the largest family. Interestingly, although *S. apiana* had more abundant ATD metabolites than that in other *Salvia* plants, CYP76AK and CYP76AH subfamilies did not seem to have experienced gene expansion, suggesting a functional diverse of these CYPs. On the other hand, we found that CYP98A subfamily might expanded, consisting with more complex composition of PAs in *S. apiana*.

Apart from CYP98A-mediated hydroxylation, polymerization of phenolic polymers promotes the metabolic variety of phenolic acids, which were previously supposed to be catalyzed by the laccase family in *Salvia* plants. However, the specific laccase remains to be identified (Di et al., 2013; Li et al., 2019; Zheng et al., 2021). Metabolic profiling revealed the absence of these phenolic polymers, implying the lack or mutation of corresponding laccases in *S. apiana*. Thus, the phylogeny of laccase family was examined in both the *S. apiana* (27 members) and *S. miltiorrhiza* (29 members) (Figure 7C). All the laccases were classified into seven clades, but clade VI was absent in *S. apiana*. We also found that the clade VII in *S. miltiorrhiza* showed significant gene expansion. Taken together, these extra laccase genes might contribute to the formation of phenolic polymers such as salvianolic acid B in *S. miltiorrhiza*.

## Discussion

*S. apiana*, known as white sage, is of ritual meaning and great medicinal value in Southern California historically. However, previous studies on *S. apiana* were mainly focused on the pharmaceutical perspective as phytochemistry and biological activities. Here, based on the establishment of full-length transcriptome of *S. apiana*, we were able to investigate its taxonomical position, chemical specificities, as well as genetic characteristics.

The origins of *Salvia* taxa in the New World, as well as their dispersal, are still debated (Will and Claßen-Bockhoff, 2017). According to the phylogenetic relationship of *S. apiana, S. splendens*, and *S. miltiorrhiza*, we estimated that the divergence between America *Salvia* taxa and North Asia taxa was at about 28.24 Mya. This was a little earlier than the previous estimation (17.6 Mya) according to the phylogeny between *Salvia bowleyana* (*S. bowleyana*), *S. splendens*, and *S. miltiorrhiza* (Zheng et al., 2021). However, both results suggested that the two lineages were diverged during late Oligocene or early Miocene, which is consisted with previous studies of the *Salvia* lineages (Kriebel et al., 2019; Krol et al., 2021). However, *S. apiana* had not been considered yet, which added nuclear gene information for North America taxa.

Besides phylogenetic position, *S. apiana* appeared to occupy a unique position in the terpenoids biosynthesis metabolic divergence. Based on the metabolic profile analyses, the presence of most of the structures of ATDs seems to be species or lineage specificity, such as tanshinones in the roots of *S. miltiorrhiza* and carnosic acid in *S. officinalis* (Guo et al., 2016; Ignea et al., 2016; Scheler et al., 2016). The production of two classes of ATDs appears to be species or taxonomy specific in such *Salvias*. Since *S. apiana* has all-around catalytic capacity for ATDs, it is supposed to be a suitable model for studying ATD biosynthesis. First, the diverse biosynthesis of different ATDs represented a typical evolutionary event for emergence and loses of metabolic pathway that accompany with speciation within the taxa (Mint Evolutionary Genomics Consortium, 2018; Lichman et al., 2020b). Second, the divergent biosynthesis routes provide direct gene information for elucidating ATD biosynthesis pathways. Based on the current understanding of ATD biosynthesis, two CYP subfamilies, CYP76AKs and CYP76AHs, are critical for the metabolic diversity of ATDs. CYP76AK6, 7, and 8 catalyze successive oxidations at C20 to generate a carboxyl group (Ignea et al., 2016; Scheler et al., 2016), whereas CYP76AK1 only generates an alcohol group at this position (Guo et al., 2016). From our transcriptome of *S. apiana*, two CYP76AKs and two CYP76AHs were discovered. We also found that CYP76AKs and CYP76AHs subfamilies in *S. apiana* were not expanded during evolution, implying that these CYPs have a broad catalytic capability. Thus, further functional investigations and whole-genome duplication (WGD) analyses of *S. apiana* genome might improve the understanding of ATDs biosynthesis in plant and the *S. apiana*'s all-around catalytic capacity. A recently published genome of *Salvia hispanica* (*S. hispanica*), which belongs to America linage as well, revealed the complexities for the genome evolution within the *Saliva* linages, as well as the evolution of ATDs biosynthesis. The size of *S. hispanica* genome is about 347.6 Mb, which is nearly half the size of other known *Salvia* genomes, as *S. miltiorrhiza* (641 Mb), *S. splendens* (711 Mb), and *S. bowleyana* (462 Mb) (Song et al., 2020; Jia et al., 2021; Zheng et al., 2021). Compared with *S. splendens*, a recent WGD was absent, thus resulting in the small size of *S. hispanica*. The WGD events during the genome evolution are always been considered as major driven forces for the chemodiversity of plants (Lichman et al., 2020a). Thus, the WGD analyses and the genome of *S. apiana* might help to explain the functional diversities of CYP76AKs and specialized ATDs production in this species. Furthermore, we believe that a lineage- or genus-wide chemical profile of ATDs would be helpful to clarify the metabolic distribution among the *Salvia* species and provide definitive evidences for fully elucidating ATDs biosynthesis.

In contrast to ATDs, phenolic polymers as salvianolic acid B were not produced by *S. apiana*, indicating the lack of corresponding catalytic enzymes (Di et al., 2013; Hou et al., 2013). The biosynthesis of phenolic acids is composed of the general phenylpropane pathway, tyrosine-derived pathway, and additional enzymes (Petersen and Simmonds, 2003). A BAHD acyltransferase member [rosmarinic acid synthase (RAS)] and CYP98A subfamily were reported to contribute to the specific PAs biosynthesis in Lamiaceae by producing rosmarinic acid. Inferring from the indirect evidences, laccase family might catalyze further polymerization (Di et al., 2013; Li et al., 2019; Zheng et al., 2021; Zhou et al., 2021); however, it still needs to be proved. The absence of salvianolic acid B in *S. apiana* implies the lose function of laccase family, which can be considered as a natural mutant. When comparing the laccase family between *S. apiana* and *S. miltiorrhiza*, we discovered that clade VII has expanded in *S. miltiorrhiza*, which included the previously predictions according to mutant experiment of *SmLAC7* and *SmLAC20*. It shows that the range of candidate genes can be effectively narrowed through the comparison of laccase families between *Salvia* species.

## Conclusion

In summary, we reported the first full-length transcriptome of medicinal plant *S. apiana*. The distribution and contents of ATDs and PAs exhibited tissue-specific patterns (leaf and root) in *S. apiana* by UPLC-MS analysis. Such tissue-specific accumulation of ATDs and PAs was further verified with genes in ATDs and PAs biosynthetic pathways through systematic metabolomic and transcriptomic analyses. Moreover, phylogenetic analysis identified five candidate genes for ATDs synthesis; further functional studies of these genes are needed to verify their roles in ATDs biosynthesis. Together, this study provides novel insights into the molecular basis for the biosynthesis of ATDs and PAs in *S. apiana* and serves as an approach with which to analyze full-length transcriptome data and the biosynthesis process in other *Salvia* plants.

## Materials and Methods

### Plant Materials

*Salvia apiana* was planted in a greenhouse with 25°C and humidity of 40–70% at Shanghai University of Traditional Chinese Medicine for 6 months (May 2020 to November 2020). The fresh leaves and roots of *S. apiana* were harvested at vegetative stage for both the metabolic and transcriptomic analyses.

### Chemical Standards

Chemical standards, including danshensu, caffeic acid, rosmarinic acid, cryptotanshinone, sugiol, carnosic acid, tanshinone IIA, and miltirone, were purchased from Shanghai Yuanye Biotechnology Corporation Ltd. (Shanghai, China). The other chemicals and reagents were purchased as follows: acetonitrile and methanol (HPLC grade), Merck (Darmstadt, Germany); warfarin, Sigma-Aldrich (Madrid, Spain); chloroform, Sinopharm Chemical Reagent (Shanghai, China); leucine encephalin, Waters (Milfor, Massachusetts, USA); pure distilled water, Watsons water (Hong Kong, China); and formic acid (HPLC grade), Fisher Scientific (Fair Lawn, New Jersey, USA).

### Ultra-High-Performance Liquid Chromatography Coupled With Quadrupole Time-of-Flight Mass Spectrometry Analysis for the Non-targeted Metabolomics Study

Each of 8 plants was divided into roots and leaves separately, represented as eight biological replicates. All the samples were crushed into homogeneous powder. Ten milligram prepared powder was extracted with 1 ml of 70% methanol (v/v) containing warfarin (5 μg/ml) as the internal reference for 1 h in an ultrasonic bath (53 kHz, 350 W) at 4°C. After centrifuged at 12,000 g under 4°C for 30 min, the supernatant was used for UHPLC-Q-TOF-MS analysis.

Analyses were performed using the ACQUITY UPLC System (Waters) coupled with the Xevo G2-XSQTOF mass spectrometer (Waters). Briefly, samples were first separated using an ACQUITY UPLC T3 column (2.1 × 100 mm, 1.8 μm). The column temperature was constant at 40°C and the flow rate was 0.40 ml/min with an injection volume of 1.0 μl. 0.1% (v/v) formic acid/water (solvent A) and 0.1% (v/v) formic acid/acetonitrile (solvent B) were the mobile phases for gradient elution. The elution gradients were: 98–80% A over 0–7 min; 80–78% A over 7–11 min; 78–40% A over 11–20 min; 40–35% A over 20–25 min; 30–35% A over 25–28 min; 35–5% A over 28–30 min; 5–5% A over 30–33 min; and the final re-equilibration at 2% A for 5 min.

To acquire mass spectrometry data, a Xevo G2–XS with an electrospray ionization source equipped with an electrospray ionization source was used. Mass spectrometry was carried out in both the positive ion and negative ion modes under 30 V cone voltage, with the capillary voltage of 3 kV (positive ion mode) or 2.5 kV (negative ion mode), respectively. The desolvation temperature was set at 450°C with the desolvation gas flow of 600 l/h and the source temperature was set at 150°C with a cone gas flow of 50 l/h. All the data were collected in MS^E^ mode, with the parameters as follows: MS^E^ range 50–1,200 m/z, MS^E^ low energy 6 eV, and MS^E^ high energy 15–30 eV. To calibrate the instrument, a sodium formate solution (0.5 mM) was used. Leucine enkephalin was continuously acquired and used as an external standard for mass correction. All the data were viewed in MassLynx version 4.2.

### Processing of Metabolomics Data

The metabolomics data was first converted to the Analysis Base File (ABF) converter format and imported into the MS-DIAL version 4.60 software (Tsugawa et al., 2015). The parameter settings were as follows: MS^1^ and MS^2^ tolerance were 0.01 and 0.02 Da, respectively. MS^1^ mass range and MS/MS mass range were set between 100 and 1,000 with the MS/MS abundance cutoff at 800 amplitudes. Retention time range was set between 1 and 28 min with the retention time tolerance at 0.15 min. Mass slice width was 0.1 Da. For negative ion mode, adduct types, including [M-H]^−^, [M+HCOO]^−^, [M+Na-2H]^−^, [M+K-2H]^−^, [2M-H]^−^, and [2M+FA-H]^−^, were selected. For positive ion mode, [M+H]^+^, [M+Na]^+^, [M+K]^+^, [M+NH4]^+^, and [2M+H]^+^ were selected. Peak tables generated from MS-DIAL were cleaned and clustered by the MS-CleanR (Fraisier-Vannier et al., 2020) with the following parameters: minimum blank ratio at 0.8; relative mass defect between 50 and 7,000 with the maximum mass difference at 0.005 Da; and maximum relative SD at 30 with the maximum retention time difference at 0.03 min. To generate the final peak table for metabolite identification, the highest intensity in each cluster was retained.

### Metabolite Identification and Chemometric Analysis

MS-FINDER was applied to identify the structures of unknown metabolites with reference databases such as UNPD, Northern African Natural Products Database (NANPDB), Human Metabolome Database (HMDB), PlantCyc, KNApSAcK, and LipidMaps (Tsugawa et al., 2016). The structures of compounds were confirmed by matching with theoretical data or public databases.

To obtain normalized metabolomics data, the raw data was processed in MS-DIAL with warfarin (5 μg/ml) as internal standard in each sample. Normalized metabolomics data was directly imported into SIMCA-P 14.1 (Umetrics AB, Umea, Sweden). After Pareto scaling, the orthogonal partial least-squares discriminant analysis (OPLS-DA) was applied to determine the supervised pattern recognition among the defined groups. To determine the unsupervised pattern recognition of leaves and roots, the principal component analysis (PCA) was performed. Only metabolites with variable importance in projection (VIP) value larger than 1.5 obtained from the OPLS-DA model were considered as the potential chemical markers with high contribution.

### Ribonucleic Acid Extraction, Quality Assessment, and Quantification

Total RNA was extracted using the TransZol Plus RNA Kit (TransGen, China) according to the manufacturer's protocol. RNA integrity was determined by the 2100 Bioanalyzer (Agilent Technologies Incorporation, Santa Clara, California, USA) and the OD260/280 values were determined with the ND-2000 (NanoDrop Technologies, Wilmington, Delaware, USA). Only high-quality RNA samples were preserved for later Iso-Seq library construction.

### PacBio Library Construction and Sequencing

The PacBio libraries were prepared separately using mixed messenger RNA (mRNA) from three leaf or root samples. The Iso-Seq library was prepared using the Clontech SMARTer PCR cDNA Synthesis Kit (Takara Bio, Mountain View, California, USA) and the BluePippin Size Selection System protocol as described by Pacific Biosciences (PN 100-092-800-03). Then, sequencing was performed on a PacBio Sequel platform (Pacific Biosciences).

### Illumina Complementary DNA Library Construction and Next-Generation Sequencing

The complementary DNA (cDNA) library for next-generation sequencing (NGS) was prepared by using the Illumina TruSeq^TM^ RNA Sample Preparation Kit (Illumina, San Diego, California, USA) according to the protocol provided by the manufacturer. Sequencing was performed on the Illumina NovaSeq 6000 Sequencer (Illumina, San Diego, California, USA) by Shanghai Majorbio Biopharm Biotechnology Corporation Ltd. (Shanghai, China).

### Sequence Assembly and Gene Annotation

The raw paired-end reads were trimmed and quality controlled by fastp (https://github.com/OpenGene/fastp) with default parameters. Then, clean data from the samples of *S. apiana* was *de-novo* assembly into contigs using Trinity assembler (Grabherr et al., 2011).

To annotate the gene function, all the assembled transcripts were searched against the following databases: National Center for Biotechnology Information (NCBI) non-redundant (NR) protein sequences database; protein family (Pfam); Clusters of Orthologous Groups of proteins (KOG/COG); a manually annotated and reviewed protein sequence database (Swiss-Prot); KEGG Ortholog database (KO); and the Gene Ontology (GO). BLASTX was used for NR, KOG, Swiss-Prot, and the KEGG database analysis with the E-value set at 1e-5 (Camacho et al., 2009). The Pfam search results were generated using HMMER version 3.1b2 (with E < 1e-5).

### Phylogenetic Tree Construction and Divergence Time Estimation

A single-copy orthologous nuclear gene set for Lamiaceae was used to construct the phylogenetic tree (Mint Evolutionary Genomics Consortium, 2018). Phylogenetic relationship among these 5 plant species was resolved using the RAxML package (version 8.1.13) (Stamatakis, 2014). Their divergence times were estimated by the program MCMCtree in PAML (version 3.15) (http://abacus.gene.ucl.ac.uk/software/paml.html).

Multiple sequence alignments of CYP targets were performed using the ClustalW program and phylogenetic trees were constructed by MEGA version 7.0. The maximum likelihood statistical method was used to calculate the phylogenetic tree, with 1,000 bootstrap replications.

### Differential Expression Analysis and Functional Enrichment

The expression level of each transcript was analyzed by using the transcripts per million reads (TPM) method in RSEM software (Li and Dewey, 2011). The analysis of differential expressed genes (DEGs) was performed by using DESeq2 (Love et al., 2014) with *p*-adjust <0.05 and |log2FC| ≧ 1. In order to identify DEGs, the GO and the KEGG enrichment analyses were performed by Goatools (https://github.com/tanghaibao/Goatools) and KOBAS (Xie et al., 2011), respectively, with significantly enriched in the GO terms and metabolic pathways at Bonferroni-corrected *P* ≤ 0.05.

## Data Availability Statement

The datasets presented in this study can be found in online repositories. The names of the repository/repositories and accession number(s) can be found below: https://ngdc.cncb.ac.cn/, CRA006765 and CRA006779.

## Author Contributions

WC and QL were the leading investigators of this research program. QL designed the experiments. JH performed most of experiments and analyzed the data. Other authors assisted in experiments and discussed the results. JH and FL wrote the manuscript. All authors contributed to the article and approved the submitted version.

## Funding

This study was financially supported by the National Key R&D Program of China (2019YFC1711100), the National Natural Science Foundation of China (32070327 and 31770329), the Young Elite Scientists Sponsorship Program by Cast (2021-QNRC1-03), and the Research Project of Science and Technology Commission of Shanghai Municipality (21DZ2202300).

## Conflict of Interest

The authors declare that the research was conducted in the absence of any commercial or financial relationships that could be construed as a potential conflict of interest.

## Publisher's Note

All claims expressed in this article are solely those of the authors and do not necessarily represent those of their affiliated organizations, or those of the publisher, the editors and the reviewers. Any product that may be evaluated in this article, or claim that may be made by its manufacturer, is not guaranteed or endorsed by the publisher.
