# Supplementary material for: Identification of Abietane-Type Diterpenoids and Phenolic Acids Biosynthesis Genes in Salvia apiana Jepson Through Full-Length Transcriptomic and Metabolomic Profiling
Source: Front Plant Sci. 2022 Jun 8;13:919025. doi: 10.3389/fpls.2022.919025 (PMC9213684; doi:10.3389/fpls.2022.919025)
Supplement: Supplementary file 1 [file Data_Sheet_1.pdf]

Supplementary Figures

Supplementary Figures

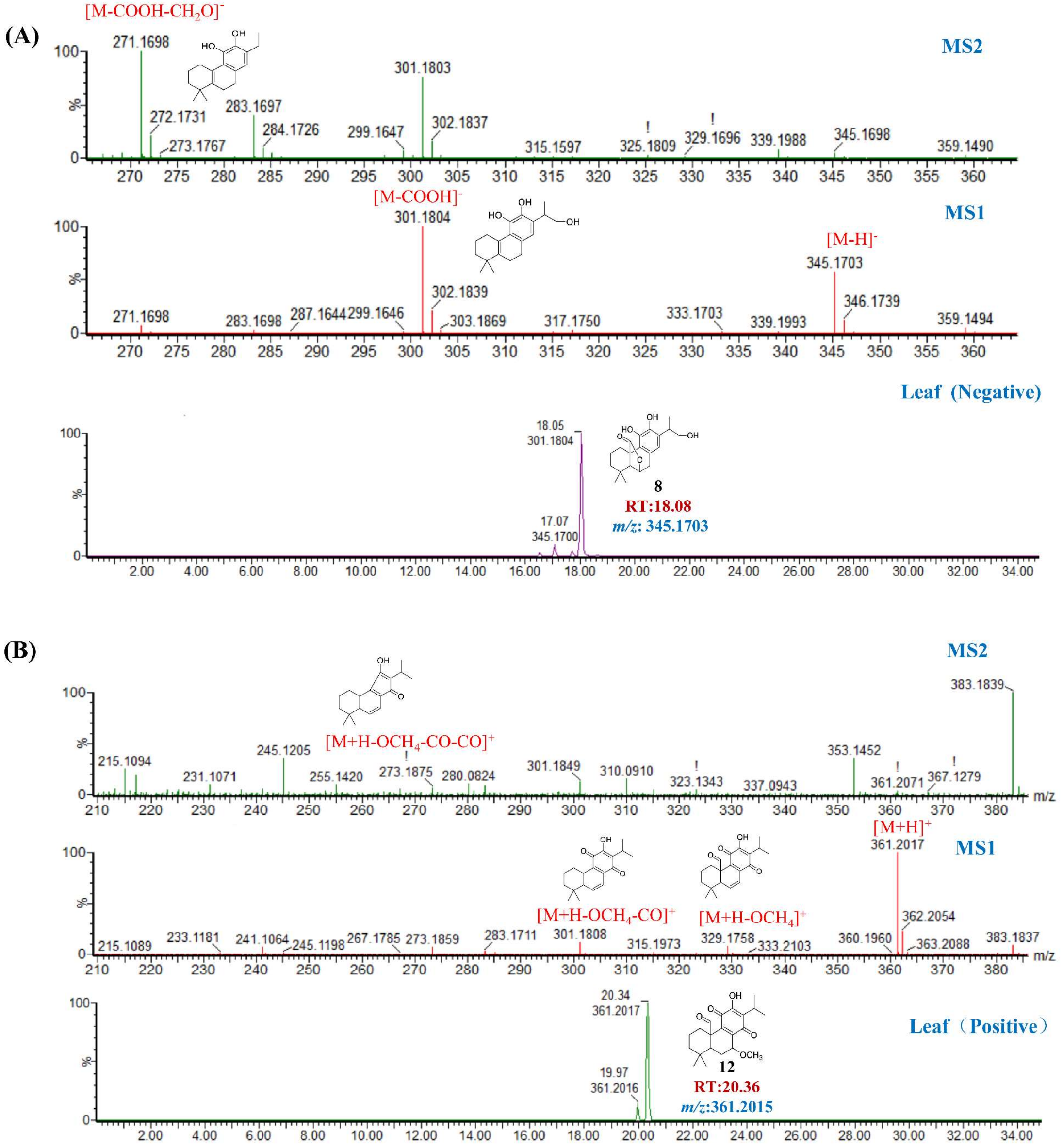

Supplementary Figure 1. The MS/MS fragment patterns of compound 9 and 12.(A)MS/MS fragment patterns of compound 9 in both negative-mode. (B) MS/MS fragment patterns of compound 12 in positive-ion mode. Compounds 9 and 12 are examples of all compounds.
